# Epiploic appendagitis – clinical characteristics of an uncommon surgical diagnosis

**DOI:** 10.1186/1471-2482-7-11

**Published:** 2007-07-01

**Authors:** Michael Sand, Marcos Gelos, Falk G Bechara, Daniel Sand, Till H Wiese, Lars Steinstraesser, Benno Mann

**Affiliations:** 1 Department of General and Visceral Surgery, Augusta Krankenanstalt, Academic Teaching Hospital of the Ruhr-University Bochum, Germany; 2 Department of Dermatology and Allergology, Ruhr-University Bochum, Germany; 3 Department of Physiological Science, University of California, Los Angeles, USA; 4 Department of Radiology, Augusta Krankenanstalt, Academic Teaching Hospital of the Ruhr-University Bochum, Germany; 5 Department of Plastic Surgery, Ruhr-University Bochum, Germany

## Abstract

**Background:**

Epiploic appendagitis (EA) is a rare cause of focal abdominal pain in otherwise healthy patients with mild or absent secondary signs of abdominal pathology. It can mimick diverticulitis or appendicitis on clinical exam. The diagnosis of EA is very infrequent, due in part to low or absent awareness among general surgeons. The objective of this work was to review the authors' experience and describe the clinical presentation of EA.

**Methods:**

All patients diagnosed with EA between January 2004 and December 2006 at an urban surgical emergency room were retrospectively reviewed by two authors in order to share the authors' experience with this rare diagnosis. The operations were performed by two surgeons. Pathological examinations of specimens were performed by a single pathologist. A review of clinical presentation is additionally undertaken.

**Results:**

Ten patients (3 females and 7 males, average age: 44.6 years, range: 27–76 years) were diagnosed with symptomatic EA. Abdominal pain was the leading symptom, the pain being localized in the left (8 patients, 80 %) and right (2 patients, 20%) lower quadrant. All patients were afebrile, and with the exception of one patient, nausea, vomiting, and diarrhea were not present. CRP was slightly increased (mean: 1.2 mg/DL) in three patients (33%). Computed tomography findings specific for EA were present in five patients. Treatment was laparoscopic excision (n = 8), excision via conventional laparotomy (n = 1) and conservative therapy (n = 1).

**Conclusion:**

In patients with localized, sharp, acute abdominal pain not associated with other symptoms such as nausea, vomiting, fever or atypical laboratory values, the diagnosis of EA should be considered. Although infrequent up to date, with the increase of primary abdominal CT scans and ultrasound EA may well be diagnosed more frequently in the future.

## Background

Epiploic appendages, also referred to as Appendices epiploicae, are between 50–100 fatty appendages originating in two rows (anterior and posterior) parallel to the external surface of the three longitudinal muscle bands of the large intestine known as taenia coli. First anatomically described in 1543 by Vesalius, they were not given any surgical significance until 1853 when Virchow suggested that their detachment might be a source of free intraperitoneal bodies [1,2].

Epiploic appendages are between 0.5 and 5 cm long, each accompanied by one or two arterioles and a venule which is present in its vascular stalks attached to the colon [3]. Torsion of the epiploic appendages is rare, but can result in ischemia presenting as an acute clinical condition which can mimic diverticulitis, appendicitis, or other more serious causes of acute abdominal pain [4]. Besides torsion, which is most likely the main pathophysiological mechanism, spontaneous venous thrombosis of an appendageal draining vein is another uncommon cause of primary epiploic appendagitis [5,6]. The sigmoid colon and the caecum are the predominant physiological sites of appendageal occurrence. However, the sigmoid colon is more frequently affected than the caecum [7]. Anatomically, the pain is therefore usually located in the left, sometimes in the right lower abdominal quadrant. Due to the lack of pathognomic clinical features the diagnosis of epiploic appendagitis is difficult. It is also very infrequent, causing awareness among general surgeons for this clinical condition to be missing sometimes.

In this study a retrospective chart review was performed in order to share the authors' experience with this rare diagnosis. A review of its clinical presentation is also given.

## Methods

This investigation originates from an urban academic surgical emergency room with approximately 8,000 annual visits. All patients diagnosed with epiploic appendagitis, either at the surgical emergency room or at discharge from the hospital, were identified by a review of the visit logs by means of an electronic patient medical record system (Care Center, V 14.0.100 Siemens Medical Solutions Health Services GmbH, Erlangen, Germany). All patients between January 2004 and December 2006 were included in the study. Informed consent was obtained from the study subjects. The retrospective chart review was performed by two authors (MS, MG). The notes of the surgical resident and the senior attending surgeon who were in care of the patient were analyzed for significant findings as anorexia, nausea, vomiting, local tenderness, site of tenderness, rebound, CT scan results, laboratory findings and results of sonography. All patients were contacted after 12 months regarding the recurrence of EA. The collected data were compiled in an electronic database (Microsoft Excel for Windows, Microsoft Corp., Redmond, WA), mean values for numeric items were calculated and data was evaluated.

## Results

Between January 2004 and December 2006 ten patients (3 females and 7 males, average age: 44.6 years, range: 27–76 years) were diagnosed with symptomatic epiploic appendagitis (EA). Abdominal pain was the leading symptom, being localized in the left (8 patients) and right (2 patients) inferior quadrant. The pain was described as a sharp localized pain which was pointed out by the patient with one finger. Its character was described as constant, ranging between 6–8 on a visual analogous pain scale (VAS; scaled 1 – 10). Patients waited 2.3 days on average (range: 1–4 days) before seeking medical attention. All the patients were afebrile and, with one exception, nausea, vomiting, and diarrhea were not present. There was no palpable mass in any patient. Rebound tenderness was found in one patient. The tentative diagnosis after medical history and physical examination was diverticulitis (n = 5), appendicitis (n = 2), neoplasma of unknown origin (n = 1) and EA (n = 2).

Laboratory analysis showed no significant findings regarding leukocyte count. However, three of ten patients (33%) in this study showed a slight increase of CRP (C-reactive protein). Maximum CRP was 1.5 MG/DL (range 0.1–1.5 MG/DL).

Sonography findings specific for EA were present in three patients (33%). Six patients received an abdominal computer tomography scan (CT scan) which showed an oval lesion less than 4 cm (mean: 2,7 cm, SD 1,49 cm, 95%CI, 1,3–4,1 cm) in diameter with an attenuation equivalent to that of fat. Additional surrounding inflammatory changes were present in two cases. Ultrasonography showed a non-compressible, hyperechoic, solid mass (n = 3). A small hypoechoic rim was described in two patients additionally to the latter findings.

Treatment was surgical (n = 9) and conservative (n = 1). Surgical therapy was performed via laparoscopy and subsequent excision of the inflamed epiploic appandage (n = 6), conventional laparotomy with simultaneous oncologic ovarectomy (n = 1) and conservative therapy with anti-inflammatory medication (n = 1). After 12 months all patients reported that there was no recurrence after treatment.

## Discussion

Epiploic appendagitis is a term introduced by Lynn et al. in 1956 and describes an uncommon diagnosis which is associated with rapid onset of localized left or right lower quadrant pain [8]. Due to the lack of pathognomonic clinical features, the diagnosis is difficult. In the following paragraphs we would like to summarize the findings of this study and aid diagnosis with additional information given.

### Anatomy

Epiploic appendages are 1–2 cm thick and 0.5–5 cm long, each supplied by one or two small colonic end-arteries and a small draining vein [3]. They are described as small, physiologic peritoneal fat pouches which are attached to the external surface of the colon by vascular stalks. The predominant localization in a series of ten patients described in this study was the sigmoid colon (n = 8). They originate next to the anterior and the posterior taenia coli predominantly at sigmoid colon and the caecum. In our study, the descendo-sigmoidal junction was the most frequent localization (33 %). One patient had a caecal EA associated with pain in the right iliac fossa. Right iliac fossa pain, however, in patients with EA does not necessarily mean that the caecum is the anatomic localization. In our study, one patient had an EA on a massive elongated sigmoid colon reaching to the right iliac fossa where the pain was located.

In 1853, Virchow suggested that a detachment of the epiploic appendages might be a source of loose intraperitoneal bodies [1]. In fact, the infarcted tissue can calcify, appearing in the abdominal cavity as "peritoneal loose body" or "peritoneal mice" incidentally found during laparoscopy or during radiologic evaluation [2]. In rare cases it might re-attach to a surface such as the lower aspect of the spleen and is then called a "parasitized appendix epiploica" [9].

### Physiology and Pathophysiology

**Figure 1:**
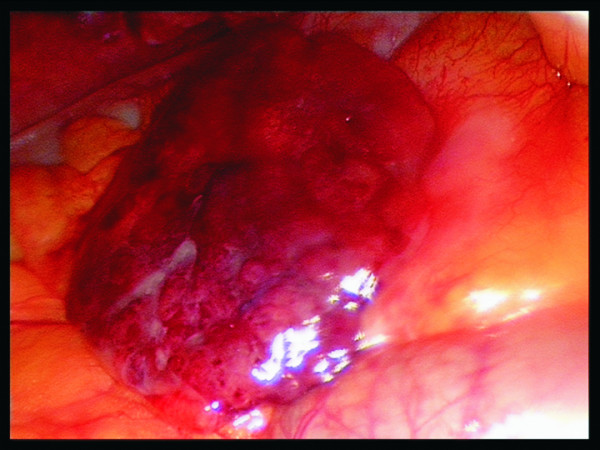
Laparoscopic view of a necrotic haemorrhagic epiploic appendage.

A variety of physiological functions of epiploic appendages have been proposed. They include the role of a soft and flexible support cushioning the colon, a role in immune response (like a small omentum) and colonic absorption [2]. Pathophysiologically a twisting, kinking or stretching of epiploic appendages along their long axis with impairment of vascular supply, subsequent venous thrombosis and necrosis is the pathophysiological sequence which, depending on localization and severity, can mimic a variety of underlying causes of abdominal conditions [10-12]. The necrosis can sometimes be haemorrhagic as shown in Fig 1. The actual torsion itself is seldomly seen at operation [13]. In our series one patient showed an EA with the actual torsion visible at the operative field. Although less likely, a primary thrombosis (de novo) without previous torsion is also conceivable.

A variety of complications can follow EA [14,15]. Accompanying surrounding inflammation can trigger adhesions with multiple secondary symptoms. Another possible complication is local abscess formation, simulating a neoplastic lesion. Intussusception, bowel obstruction, abscess formation and peritonitis are further described complications which were not found in our group of patients [7]. The rare case of simultaneous epiploic appendagitis and a neoplastic lesion was found in patient number 6 whose bilateral ovarian cancer was diagnosed during radiologic work up of left lower quadrant pain due to a sigmoidal epiploic appendagitis (Tab 1). Although very rare, peritoneal loose bodies have been described as cause for intestinal obstruction or urine retention, depending on their size and intra-abdominal localization [16,17].

**Table 1:** Clinical data of ten patients with epiploic appendagitis

**Patient Number**	**Sex**	**Age**	**Site**	**Temperature**	**WBC (4.4–11.3/NL)**	**CRP (< 0,5 MG/DL)**	**Radiologic Diagnosis CT-Scan**	**Radiologic Diagnosis Sonography**	**Duration of Pain (days)**	**Symptoms typically for**	**Operative diagnosis**	**Size (cm)**	**Localisation**	**Treatment**
1	F	42	RLQ	37.5	9000	0.1	EA	EA	2	Acute appendicitis	EA	2 × 1.5	Caecum	Laparoscopic Excision of the EA
2	M	35	LLQ	37	7200	0.1	NPF	NPF	1 (recurrent pain one month before for 2 days)	Diverticulitis	EA	2 × 3.5	Upper Sigmoid Colon	Laparoscopic Excision of the EA
3	M	52	RLQ	37.1	10.400	1.3	n.a.	NPF	4	Acute appendicitis	EA	2.5 × 5	Sigmoid Colon (Massive Elongated) reaching to the RIF	Laparoscopic Excision of the EA
4	F	76	LLQ	37.0	5700	1.5	Tumorlike thickening of the sigmoid colon wall, fat isodense, Liposarcoma DD: EA	NPF	3	Diverticulitis	Adhesions of fat to the left inferior abdominal wall, EA torsion between the Adhesions	2 × 4	Upper Sigmoid Colon	Laparoscopic Excision of the EA
5	M	40	LLQ	37.3	11.400	0.2	EA	NPF	2 (recurrent pain one month before for 3 days)	Diverticulitis	Adhesions of the distal Colon descendens to the abdominal wall. EA torsion between the Adhesions.	1 × 1	Upper-Sigmoid Colon	Laparoscopic Excision of the EA
6	F	75	LLQ	37.1	6200	0.4	Bilateral Ovarial Cancer	Abdominal mass	3	Bilateral Ovarial Cancer	Ovarian Cancer and EA	2 × 1.5	Sigmoid Colon	Excision of the Ovarial Cancer and the EA
7	M	34	LLQ	37.2	9400	0.9	EA	EA	2 (recurrent pain one month before for 1 day)	Diverticulitis	EA	n.a.	Sigmoid Colon	Laparoscopic Excision of the EA
8	M	27	LLQ	36.9	8300	0.5	n.a.	EA	1	EA	n.a.	2.5 × 4	n.a.	Conservative Therapy
9	M	28	LLQ	37.3	10900	0.5	EA	NPF	2 (recurrent pain one and two months before for 2 days)	EA	EA	1.5 × 4.5	Upper Sigmoid Colon	Laparoscopic Excision of the EA
10	M	37	LLQ	37.2	7600	0.4	EA	NPF	3	Diverticulitis	EA	2 × 3	Descending Sigmoid Colon	Laparoscopic Excision of the EA

Legend: Site = site of the pain; WBC = white blood cell count prior to surgery; EA = Epiploic appendagitis; RLQ = right lower quadrant; LLQ = left lower quadrant; NPF = no pathological finding; n.a. = not available

### Clinical Characteristics

Epiploic appendagitis can occur at any age. In our study the mean age was 44.6 years with a range from 27–76 years. The reported ages range from 12 to 82 years [2]. Men are slightly more affected than women (7 male vs. 3 female in our study) which has also been confirmed by other authors [18].

On clinical exam patients describe a localized, strong, non-migratory, sharp pain which usually started after a specific physical movement of their body like postprandial exercise. An abdominal tenderness was present in all patients. In our series, patients otherwise felt healthy and rarely described other symptoms. There is a lack of fever, vomiting or leukocytic response. With diverticulitis and appendicitis being the most important causes of lower abdominal pain, they are the most frequent clinical diagnosis before radiologic imaging or diagnostic laparoscopy. The pain usually is on the left or right lower abdominal quadrant [19]. When it is on the right lower abdominal quadrant it may mimic acute appendicitis, but more often it is present on the left side mimicking acute sigmoid diverticulitis. As stated in a previous study by Son et al. we were also not able to find an association between obesity and EA which has been previously reported (p > 0,05) (8,20). The mean body mass index was 27.2 kg/m^2 ^(25–31 kg/m^2^). Laboratory values were within normal limits with exception to CRP which was slightly increased (between 1–2 mg/DL) in 2 patients (25%). It has been hypothesized that necrosis is a strong stimulus for CRP increase in patients suffering from myonecrosis [21]. It is possible that ischaemic fat necrosis like in epiploic appendagitis might trigger an inflammatory response, which – similar to myonecrosis – results in the slight increase of CRP value.

### Radiologic Evaluation

In the past, diagnosis of EA was often the result of an unexpected finding at exploratory laparotomy. Today, however, a variety of ultrasound (US) and CT findings has been described which in some cases aids the surgeon to make the right diagnosis pre-operatively. US sometimes shows an oval, non-compressible hyperechoic mass with a subtle hypoechoic rim directly under the site of maximum tenderness [22-24]. There is no central blood flow depicted on color Doppler US imaging [22]. The latter described picture was observed in 3 of 10 patients (33%). Ultrasound in combination with CT scan made the correct preoperative diagnosis possible in 3 of 10 patients (Tab 1).

**Figure 2:**
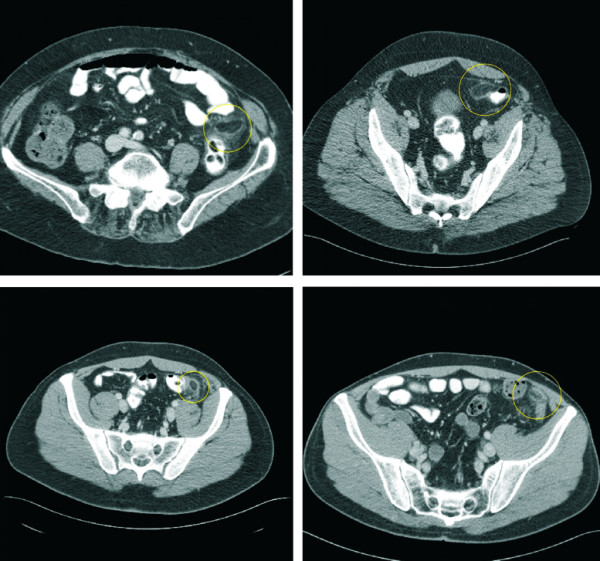
Abdominal CT scan demonstrating the horizontal section of four different patients with EA (circle).

Normal epiploic appendages are not seen on CT scan. They typically have fat attenuation and cannot be distinguished from other adipose structures like retroperitoneal fat unless they are surrounded by intraperitoneal fluid or inflammation. With the introduction of cross-sectional imaging and the increasing use of abdominal CT scan for primary evaluation of lower abdominal pain the recognition of EA has increased. In 1986 Danielson et al. were the first that reported an EA diagnosed by CT scan. Meanwhile this entity has become more common and numerous reports describing CT scan features have been published [23-25]. Pathognomonic CT scan findings are a 2–4 cm, oval shaped, fat density lesion, surrounded by inflammatory changes (Fig 2 + 3). One can distinguish a central focal area of hyper-attenuation with surrounding inflammation. Thickening of the parietal peritoneum wall can be sometimes observed. In contrast to diverticulitis the diameter of the colonic wall is mostly regular without signs of thickening.

**Figure 3:**
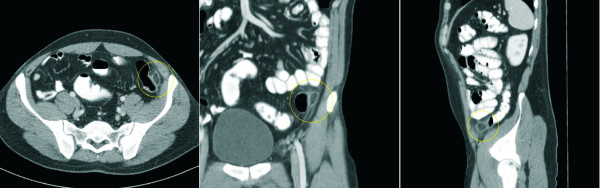
Abdominal CT scan demonstrating the horizontal, coronal and sagittal section of a patient with EA (circle).

### Therapy

Therapy of epiploic appendagitis is a topic of some controversy. It is described by some authors as a self-limiting condition with patients recovering in less than 10 days with oral anti-inflammatory medication [22]. Most of the surgical literature underlines the benign course of disease and favors a conservative therapy regiment. This is a widely applied form of therapy which is practiced with success. However, we have observed that there is a tendency of recurrence in conservatively treated patients. Four of ten patients in this study (40%) already had the same pain at the same localization, for two days on average, four weeks before presenting to the emergency department. This cannot be taken as decisive evidence that EA recurs if not treated surgically. Nevertheless it arouses suspicion that conservative forms of therapy might have a tendency for recurrence and surgical interventions should be considered. In the authors' personal opinion surgical therapy is favorable to prevent recurrence, inflammation induced adhesions and other less common complications. Laparoscopic interventions are highly appealing to both patient and surgeon. We favor surgical exploration via a laparoscopic approach with simple ligation and excision of the inflamed appendage. Patients recover fast and can quickly return to work. In addition, the radiation exposure by the use of multiple follow up CT scans in otherwise mostly healthy, young individuals has to be considered when discussing surgery via a laparoscopic approach as a curative form of therapy. On the other hand every unnecessary surgery has to be prevented. The usual main complications of surgery as excessive bleeding, infection or an unexpected reaction to the anaesthetic as well as specific complications of laparoscopy like accidental damage to internal organs or abdominal bruising are down sides of the surgical approach and have to be discussed with the patient. Limitation of the study is the small sample size. A final conclusion regarding the best form of therapy cannot be drawn. We propose that a study evaluating a larger number of patients (possibly with a group of conservatively treated patients) may be of interest to further evaluate this controversy issue.

## Conclusion

Epiploic appendagitis is a surgical diagnosis with clinical features that may guide the surgeon to the right pre-operative diagnosis. In patients with localized, sharp, acute abdominal pain which is not associated with other symptoms like nausea, vomiting, fever or typical abdominal laboratory values, the diagnosis of EA should be considered as a rare differential diagnosis to sigmoid diverticulitis and appendicitis.

Although infrequent until now, with the increase of primary abdominal CT scans and ultrasound, which have become standard diagnostic imaging tools, EA will be diagnosed more frequently in the future. This study describes the clinical features of EA as a possible guide to the surgeon for the correct diagnosis of this rare disease.

## Competing interests

All authors hereby disclose any commercial associations which might pose or create a conflict of interest with information presented in this manuscript. The authors declare that they have no competing interests.

## Authors' contributions

MS: Surgeon who performed the operations, documented and prepared the draft. Performed the retrospective chart review.

MG: Surgeon who performed the operations. Performed the retrospective chart review and helped with editing of the manuscript.

FGB: Literature search, revision of bibliography. Revised and edited most of the manuscript.

DS: Literature search and edited part of the manuscript.

THW: Edited part of the manuscript and interpreted radiologic images.

LS: Literature search and edited part of the manuscript.

BM: Surgeon who performed the operations and edited part of the manuscript and helped in preparing the draft.

## Pre-publication history

The pre-publication history for this paper can be accessed here:


